# A mitochondrial HSP70 (HSPA9B) is linked to miltefosine resistance and stress response in *Leishmania donovani*

**DOI:** 10.1186/s13071-016-1904-8

**Published:** 2016-12-01

**Authors:** P. Vacchina, B. Norris-Mullins, E. S. Carlson, M. A. Morales

**Affiliations:** Eck Institute for Global Health, Department of Biological Sciences, University of Notre Dame, Notre Dame, IN 46556 USA

**Keywords:** *Leishmania donovani*, Miltefosine, Proteomics, Heat-shock proteins

## Abstract

**Background:**

Protozoan parasites of the genus *Leishmania* are responsible for leishmaniasis, a neglected tropical disease affecting millions worldwide. Visceral leishmaniasis (VL), caused by *Leishmania donovani*, is the most severe form of leishmaniasis with high rates of mortality if left untreated. Current treatments include pentavalent antimonials and amphotericin B. However, high toxicity and emergence of resistance hinder the success of these options. Miltefosine (HePC) is the first oral treatment available for leishmaniasis. While treatment with HePC has proven effective, higher tolerance to the drug has been observed, and experimental resistance is easily developed in an in vitro environment. Several studies, including ours, have revealed that HePC resistance has a multi-factorial origin and this work aims to shed light on this complex mechanism.

**Methods:**

2D-DIGE quantitative proteomics comparing the soluble proteomes of sensitive and HePC resistant *L. donovani* lines identified a protein of interest tentatively involved in drug resistance. To test this link, we employed a gain-of-function approach followed by mutagenesis analysis. Functional studies were complemented with flow cytometry to measure HePC incorporation and cell death.

**Results:**

We identified a mitochondrial HSP70 (HSPA9B) downregulated in HePC-resistant *L. donovani* promastigotes. The overexpression of HSPA9B in WT lines confers an increased sensitivity to HePC, regardless of whether the expression is ectopic or integrative. Moreover, the increased sensitivity to HePC is specific to the HSPA9B overexpression since dominant negative mutant lines were able to restore HePC susceptibility to WT values. Interestingly, the augmented susceptibility to HePC did not correlate with an increased HePC uptake. *Leishmania donovani* promastigotes overexpressing HSPA9B were subjected to different environmental stimuli. Our data suggest that HSPA9B is capable of protecting cells from stressful conditions such as low pH and high temperature. This phenotype was further corroborated in axenic amastigotes overexpressing HSPA9B.

**Conclusions:**

The results from this study provide evidence to support the involvement of a mitochondrial HSP70 (HSPA9B) in experimental HePC resistance, a mechanism that is not yet fully understood, and reveal potential fundamental roles of HSPA9B in the biology of *Leishmania*. Overall, our findings are relevant for current and future antileishmanial chemotherapy strategies.

**Electronic supplementary material:**

The online version of this article (doi:10.1186/s13071-016-1904-8) contains supplementary material, which is available to authorized users.

## Background

The intracellular protozoan parasites belonging to the genus *Leishmania* are the causative agents of leishmaniasis, a neglected tropical disease with several clinical manifestations. Species of the *Leishmania donovani* complex are responsible for the most severe form of the infection, visceral leishmaniasis (VL), also known as kala-azar [1]. VL is clinically characterized by fever, loss of appetite, fatigue, hepatosplenomegaly and splenomegaly. If VL is not treated the fatality rate can be as high as 100% in developing countries. As much as 90% of VL cases take place in Bangladesh, Brazil, Ethiopia, India, South Sudan and Sudan and approximately 500,000 new cases are reported each year [2]. *Leishmania* spp. have a digenetic life-cycle including both extracellular promastigote and obligate intracellular amastigote forms. Extracellular flagellated promastigotes reside in the midgut of the phlebotomine sand fly vector. Following infection in the mammalian host, promastigotes are engulfed by macrophages where they differentiate into non-motile amastigotes in the phagolysosome. This differentiation is triggered by environmental cues, mainly pH and temperature [3].

For the last 75 years, control of the illness has largely relied on the use of pentavalent antimonials, but resistance emergence and high toxicity have been the driving forces to seek novel and more effective treatments. For almost 15 years, the alkyl-lyso-phospholipid analogue hexadecyl-phosphatidylcholine has been used as the first and only oral drug to treat VL. This compound, commercially known as miltefosine (HePC), was initially introduced as an anti-neoplastic treatment in the 1980s, however an alternative use was discovered by Croft and collaborators in 1987 when they explored its effect against *L. donovani* amastigotes in cultured mouse peritoneal macrophages [4]. Since 1999, the use of HePC as an oral treatment has been evaluated in several clinical trials, showing 90–100% cure rate and up to 20% relapse rates [5]. While evidence of clinical HePC resistance is limited, its long half-life and low therapeutic window favour the resistance emergence.

It has been previously shown that the most common experimental mechanism of HePC resistance is associated with reduced drug accumulation. The presence of loss of function mutations in the HePC transporter subunits, MT and/or Ros3, as well as an increased efflux of the drug as a consequence of the overexpression of ABC transporters may lead to reduce HePC susceptibility [6–8]. After a comprehensive study of the phenotypical traits of clonal lines of HePC resistant *L. donovani* promastigotes, which included whole genome and RNA sequencing, we concluded that defects in drug translocation machinery cannot thoroughly explain the multifactorial mechanism of HePC resistance. Amino acid and folate metabolism, changes in membrane composition and stress-induced responses are likely involved in the resistant phenotype, as revealed by the differential abundance of these transcripts [9].

To obtain a better understanding of the complex mechanism associated with HePC resistance, we further complemented our studies with a proteomic approach and compared the protein expression profiles of in vitro HePC resistant lines to wild type *L. donovani* promastigotes. Whole protein extracts from *L. donovani* HePC resistant promastigotes were analyzed via 2D-DIGE and a mitochondrial isoform of HSP70 (LinJ.30.2480), referred to as HSPA9B [10], was identified. Heat-shock proteins (HSP) are highly conserved and constitutively expressed molecules whose chief functions are to facilitate protein synthesis and folding. Additionally, they contribute to protein assembly, intracellular trafficking, turn-over and regulation [11]. Originally discovered as gene products whose overexpression was associated to a heat-shock response, it is now widely known that their synthesis can be induced by cellular stress other than heat, such as exposure to cold, UV light, starvation, hypoxia, exposure to toxins, tissue remodeling, etc. These molecules are traditionally classified base on their molecular mass [12]. The 70-kDa family of HSPs are represented by several homologs that differ in their subcellular localization (i.e. cytoplasm, ER, mitochondria and chloroplasts). In higher eukaryotes, it is possible to find both constitutively expressed (HSC70) and stress inducible isoforms (HSP70). Mitochondrial HSP70s can be localized at the inner mitochondrial membrane or the mitochondrial matrix and phylogenetic studies indicated that this subtype is more closely related to the bacterial DnaK [13]. Similar to their cytosolic counterpart, mitochondrial HSP70s display housekeeping functions and are responsible for protein folding and assembly, refolding, organelle trafficking and quality control [11]. All of these activities require HSP70 proteins to recognize and interact with hydrophobic peptide segments in an ATP-dependent mechanism. The HSP70 family is highly conserved and each protein contains an N-terminal ATPase domain, a substrate binding domain comprising of both a 25 kDa β sheet subdomain and a 10 kDa helical subdomain and lastly a C-terminal domain rich in alpha helical structure [11, 14]. Several biochemical studies carried out in both bacteria and yeast have demonstrated the essentiality of specific amino acids localized at the ATPase and substrate binding domains for proper protein functioning [15–18]. HSP70 molecules have been widely studied in trypanosomes, as temperature shifts are part of the life-cycle of these organisms. It has been shown that a mitochondrial member of the HSP70 family in *Trypanosoma cruzi* is associated with kinetoplast DNA [19, 20] and in dyskinetoplastic *Trypanosoma brucei* mutants, the protein is distributed throughout the mitochondria [21]. Studies carried out in *Leishmania major* demonstrated that a mitochondrial HSP70 isoform is constitutively expressed and localizes to the mitochondria in all the parasite life stages [22]. The expression of a family of HSP70 genes in *L. donovani* was similarly detected in all parasite life stages but in contrast to what it was observed in *L. major*, *T. cruzi* and *T. brucei*, the expression level was not significantly heat inducible [23].

In this work, we employ quantitative proteomics and a gain-of-function approach to link HSPA9B protein to HePC susceptibility. Interestingly, this phenotype is not related to a faulty drug accumulation. Furthermore, we find transgenic parasites to be more resistant to several stress conditions, suggesting that HSPA9B contributes to the parasite stress response.

## Methods

### Parasite culture

*Leishmania donovani* wild type strain 1S2D (MHOM/SD/62/1S-CL2D), LdR10.1 and LdR30.1 clones [9] and transgenic lines were cultured in M199 supplemented with 10% FCS at 26 °C and pH 7.4 and axenic amastigotes were differentiated as described previously [24]. HePC resistant parasites were continuously maintained in the presence of 10 and 30 μM of HePC.

### Molecular constructs and parasite transfection

In order to generate a recombinant HSPA9B protein, a 2 kb region containing HSPA9B (LinJ.30.2480) was amplified from genomic DNA of *L. donovani* using the primers 5′-GCA GAT CTA TGT TCG CTC GTC GTG TGT GC-3′(BglII) and 5′-CGC GGT ACC CTT CTT TTC CTC GCT GTT CT-3′(KpnI) and LongAmp high-fidelity Taq-DNA polymerase (New England Biolabs, Ipswich, USA) following the manufacturer’s recommendation. The product was cloned into pGEM-T (Promega, Fitchburg, USA) to create pGEM-T-HSPA9B. C-terminal HSPA9B-mCherry fusion was obtained by inserting the 2 kb HSPA9B construct from pGEM-T into the respective site of pLEXSY-mCherry (HYG). The HSPA9B-mCherry-pLEXSY construct was then transformed in DH5α *E. coli* cells (New England Biolabs, Ipswich, USA). N-terminal GFP-HSPA9B fusions were obtained by inserting the 2 kb HSPA9B construct from pGEM-T into pXG-GFP + 2′ [25]. Episomal (GFP-HSPA9B) and integrative (HSPA9B-mCherry) transgenic parasites were established by electroporation of *L. donovani* promastigotes from logarithmic culture (density < 5 × 10^6^/ml) with 75 μg of circular DNA or 7.5 μg of SwaI linearized DNA, respectively. Transfected cells were grown in media containing 50 μg/ml HygromycinB (HYGB) and further selected in 100 μg/ml HYGB or selected in media containing 20 μg/ml G418. Mock transfection controls were performed in an identical manner with the respective empty vector. Generation of mutants for HSPA9B was accomplished with the QuikChange® II XL Site-Directed Mutagenesis kit (Stratagene, La Jolla, USA). Glycine 220 and 221 were mutated to Aspartic acid (G220D, G221D) and Phenylalanine 436 to Valine (F436V) to generate HSPA9B-mCherry (GxD, GxD) and HSPA9B-mCherry (GxD, GxD, FxV) mutants. Constructions were episomally introduced as described above.

### Growth curves and drug susceptibility assays

The growth rate of transfected parasite cultures was measured and compared to mock controls. Parasites were seeded in triplicate at an initial concentration of 5 × 10^5^ parasites/ml and promastigote density was assessed daily by direct microscopic counting until the parasites reached stationary phase. The leishmanicidal effect of HePC (Sigma-Aldrich, St. Louis, USA), pentamidine isethionate (Sigma-Aldrich, St. Louis, USA), amphotericin B (Sigma-Aldrich, St. Louis, USA), potassium antimony (III) tartrate hydrate (Sigma-Aldrich, St. Louis, USA) and paromomycin sulfate salt (Sigma-Aldrich, St. Louis, USA) was also evaluated. For this purpose, culture viability was measured by using the resazurin-based method CellTiter-Blue (Promega, Fitchburg, USA) as described previously [26]. Briefly, 200 μl 1 × 10^6^ parasites/ml were seeded in each well of a 96-well plate and incubated in the presence of increasing drug concentrations for 48 h at 26 °C along with appropriate solvent controls. 100 μl of each well was transferred to a new plate, 20 μl of the reagent was added and after 4 h at 37 °C fluorescence was measured (555 nm λexc/580 nm λem) using a Typhoon FLA 9500 laser scanner (GE Healthcare, Chicago, USA) and analyzed with ImageQuant TL software (GE Healthcare, Chicago, USA). Each assay was performed in triplicate. The EC_50_ values were calculated by non-linear regression analysis using SigmaPlot for Windows version 11.0.

### Determination of HePC accumulation

Parasites from a logarithmic-phase culture were washed with HPMI buffer [20 mM HEPES, 132 mM NaCl, 3.5 mM, KCl, 0.5 mM MgCl_2_, 1 mM CaCl_2_, (pH 7.4)] and pre-incubated in the same buffer for 30 min at 26 °C with 1 mM phenylmethylsulfonyl fluoride (to block lipid catabolism) and then labeled with 5 μM HePC-BODIPY [27] for 45 min at 26 °C in the dark. The fluorescent analog was added directly from an ethanol stock solution. Parasites were washed with HPMI supplemented with 4% BSA to remove the non-internalized probe (back-exchange), re-suspended in HPMI and maintained on ice. Cellular fluorescence was measured by flow cytometry using a Beckman Coulter FC500 Flow Cytometer. Propidium iodide was added to monitor cell viability and 10,000 events per sample were recorded. Solvent controls were included and the experiment was performed in triplicate.

### Indirect immunofluorescence

Cells were fixed with 4% paraformaldehyde, permeabilized with 0.1% Triton X-100, blocked with 5% BSA, and incubated with specific anti-lipoic acid antibody (Abcam, Cambridge, UK) at 1:1,000 dilution. After washing, the cells were incubated with anti-rabbit Alexa 488 conjugated antibody (1:200 dilution), washed and treated with DAPI to stain DNA. The slides were mounted with VectaShield (Vector Laboratories, Burlingame, USA). Cell fluorescence was captured with a Nikon fluorescence microscope equipped with a camera and the appropriate filters.

### Sequence alignment and bioinformatics

DNA sequence data was accessed via http://www.genedb.org. Homology searches were carried out using BLAST with the default BLOSUM-62 substitution matrix [28]. Multiple sequence alignments of HSP70 homologs were generated with ClustalX (v2.0). Alignments were converted to MEGA compatible files and fed into the MEGA 6.0 software package. A Neighbor-Joining tree was computed with 500 bootstrap replicates. The Prosite (http://prosite.expasy.org/) and Pfam (http://pfam.xfam.org/) databases were used to identify conserved domains.

### Subcellular fractioning and digitonin membrane permeabilization

For subcellular fractioning, the Qproteome Cell Compartment Kit (Qiagen, Valencia, USA) was used following the manufacturer’s specifications. Briefly, transgenic parasites from a logarithmic phase culture were washed, pelleted and resuspended in 1 ml of lysis buffer. After 10 min incubation at 4 °C, the lysate was centrifuged and the supernatant (cytosolic fraction) was recovered. The pellet was resuspended in 1 ml of extraction buffer CE2 and following a 30 min incubation period, the supernatant containing membrane proteins and proteins from the lumen of organelles (e.g. the ER and mitochondria) was recovered. Digitonin membrane permeabilization was done as previously described [29]. Monitoring of protein distribution was analyzed by western blot.

### Western blot analysis

Crude cell lysates, digitonin enriched and membrane and lumen organelle enriched fractions were separated in 4–12% Bis-Tris NuPAGE gels (Life, Carlsbad, USA) and revealed using the following antibodies: anti-GFP-horseradish-peroxidase (HRP)-conjugated antibody (Miltenyi, Bergisch Gladbach, Germany), rabbit polyclonal anti-mCherry antibody (Abcam, Cambridge, UK), mouse monoclonal anti-A2 antibody (Abcam, Cambridge, UK), mouse monoclonal anti-tubulin antibody (Sigma-Aldrich, St. Louis, USA), rabbit polyclonal anti-lipoic acid antibody (Abcam, Cambridge, UK) and anti-rabbit or anti-mouse HRP-conjugated secondary antibodies (Pierce, Waltham, USA). After washing, blots were developed using the SuperSignal chemiluminescent detection system (Pierce, Waltham, USA).

### Sample preparation and labeling for 2D- DIGE

Subcellular fractioned extracts as well as whole protein extracts from logarithmic cultures of *L. donovani* WT, LdR10.1, LdR30.1 and HSPA9B-mCherry promastigotes were precipitated using a 2-D Clean-Up kit (GE Healthcare, Chicago, USA), allowing for quantitative precipitation and removal of interfering substances such as detergents, salts, lipids, phenolics, and nucleic acid. Proteins were then differentially labelled with spectrally resolvable Cy3 and Cy5 as previously described [30]. A pool of extracts was labelled with Cy2 for normalization purposes, following the manufacturer’s recommendations (GE Healthcare, Chicago, USA).

### Isoelectric focusing (IEF) and two-dimensional gel electrophoresis (2D)

IEF of 275 μg of protein was carried out using an EttanIPGphor 3 System (GE Healthcare, Chicago, USA) at 20 °C with 13 cm non-linear DryStrip (pH 4–7). Strips were passively rehydrated overnight at room temperature in rehydration solution (GE Healthcare, Chicago, USA) containing 0.5% IPG buffer 4–7 and 2% DTT. The IEF maximum current setting was 50 mA/strip. The following conditions were programmed for IEF: 100 V gradient step for 5 h, 300 V gradient step for 5 h, 1,000 V gradient step for 2 h, 6,000 V gradient step for 8 h and 6,000 V for 5 h (60,550 Vh). Following IEF, strips were equilibrated in two different solutions (6 M urea, 75 mM Tris/HCl pH 8.8, 29.3% glycerol, 4% SDS, and 0.002% bromophenol blue) supplemented with 65 mM DTT or 13.5 mM iodoacetamide for 15 min each. The strips were transferred to SDS polyacrylamide gels and sealed with 0.5% agarose in 25 mM Tris-base, 0.19 M glycine, 0.2% SDS and 0.01% bromophenol blue. Electrophoresis was carried out in an SE 600 Ruby cooled electrophoresis system (GE Healthcare, Chicago, USA) using 12.5% SDS-PAGE gels and a two-step run (1 W/gel for 15 min and 7 W/gel for 5 h).

### Staining procedures and image analysis

After electrophoresis, gels were scanned on a Typhoon FLA 9500 Imager (GE Healthcare, Chicago, USA) using 488/520 nm for Cy2, 532/580 nm for Cy3, 633/670 nm for Cy5 and 100 mm as pixel size. Gel images were normalized by adjusting PMT voltage to obtain appropriate pixel value without any saturation. Images were analyzed with Delta2D v.4.5 software (Decodon). Gels were matched and warped and spots detected across all images. A 2-fold difference in abundance, with *P*-values < 0.05, was considered significant for the expression profiles. Polyacrylamide gels were then fixed in 50% methanol and 7% acetic acid and stained using SYPRO Ruby total protein gel stain (Life, Carlsbad, USA). Spots of interest were manually excised from gels visualized on a blue-light transilluminator (Life, Carlsbad, USA).

### Protein identification

Gel spots were subjected to reduction with 55 mM DTT in 25 mM ammonium bicarbonate (ThermoFisher Scientific, Waltham, USA) at 56 °C for 1 h followed by alkylation with 100 mM iodoacetamide (Sigma-Aldrich, St. Louis, USA) in 25 mM ammonium bicarbonate at room temperature in the dark for 45 min. The spots were washed with 25 mM ammonium bicarbonate for 10 min followed by two consecutive washes with 25 mM ammonium bicarbonate in 50/50 acetonitrile: water for 5 min, each. The spots were placed in a vacuum concentrator to dry completely before the addition of 12.5 ng trypsin gold (Promega, Fitchburg, USA) to each gel spot. The spots were kept at 4 °C for 30 min to swell and then were incubated at 37 °C overnight. Following trypsin digestion, the supernatant was collected. Peptides were further extracted from the gel spots with two consecutive additions of 50% acetonitrile/ 45% water/ 5%formic acid followed by 30 min of vortexing. The two sets of extracts were combined with the supernatant from each gel spot and then vacuum concentrated to 10 ml. Each concentrated digest was desalted with a C18 Ziptip (EMD Millipore, Darmstadt, Germany) according to the manufacturer instructions. The desalted digests were then dried down in a vacuum concentrator and reconstituted in 10 ml of 0.1% TFA in water. A 2 ml aliquot of each gel digest was injected onto a nanoAcquity UPLC (Waters Corporation, Milford, USA) with a BEH300 C18 100 mm6100 mm column (Waters Corporation, Milford, USA) with 1.7 mm particle size. A gradient of 0.1% formic acid in water (A) and 0.1% formic acid in acetonitrile (B) was performed starting with 2% B held for 6 min and then ramping to 40% B to 40 min and 90% B to 43 min. The column was washed with 90% B for 7 min and then re-equilibrated with 98% A: 2% B. The nanoAcquity was coupled to a LTQ Orbitrap Velos mass spectrometer (Thermo-Fisher Scientific, Waltham, USA) for data dependent scans of the digested samples in which the top nine abundant ions in a scan were selected for CID fragmentation. The UPLC-MS/MS chromatograms and spectra were analyzed using Xcalibur software (Thermo-Fisher Scientific, Waltham, USA), and the extracted data were searched against the *L. infantum* custom database via Mascot and/or Protein Pilot. Search criteria included a global modification of carbamidomethylation on the cysteines. Proteins identified had less than a 1% false discovery rate.

### Parasite stress response and reactive oxygen species (ROS) production

HSPA9B overexpressing promastigotes and their respective mock controls were exposed to different stress conditions: low pH (pH = 5.5), heat-shock (37 °C), heat-shock (37 °C) plus low pH (pH = 5.5), starvation and HePC treatment during 4 and 18 h. Exposure of phosphatidylserine (PS) residues was investigated with Annexin-V-FITC or Annexin-V-Alexa 594 following the manufacturer’s instructions. Propidium Iodide (Invitrogen, Waltham, USA) was added and the cells were incubated for 20 min. Analyses were performed in a Beckman Coulter FC500 Flow Cytometer. The production of intracellular ROS under the conditions described above was measured using MitoSOX Red (Molecular Probes, Eugene, USA). The intracellular fluorescence was measured by flow cytometry.

### Statistics

Significance was determined by *P*-values calculated from a two-tailed Student’s *t*-test in GraphPad Prism 7.0 unless otherwise stated.

## Results

### Identification of HSPA9B and *in silico* analysis

**Fig. 1:**
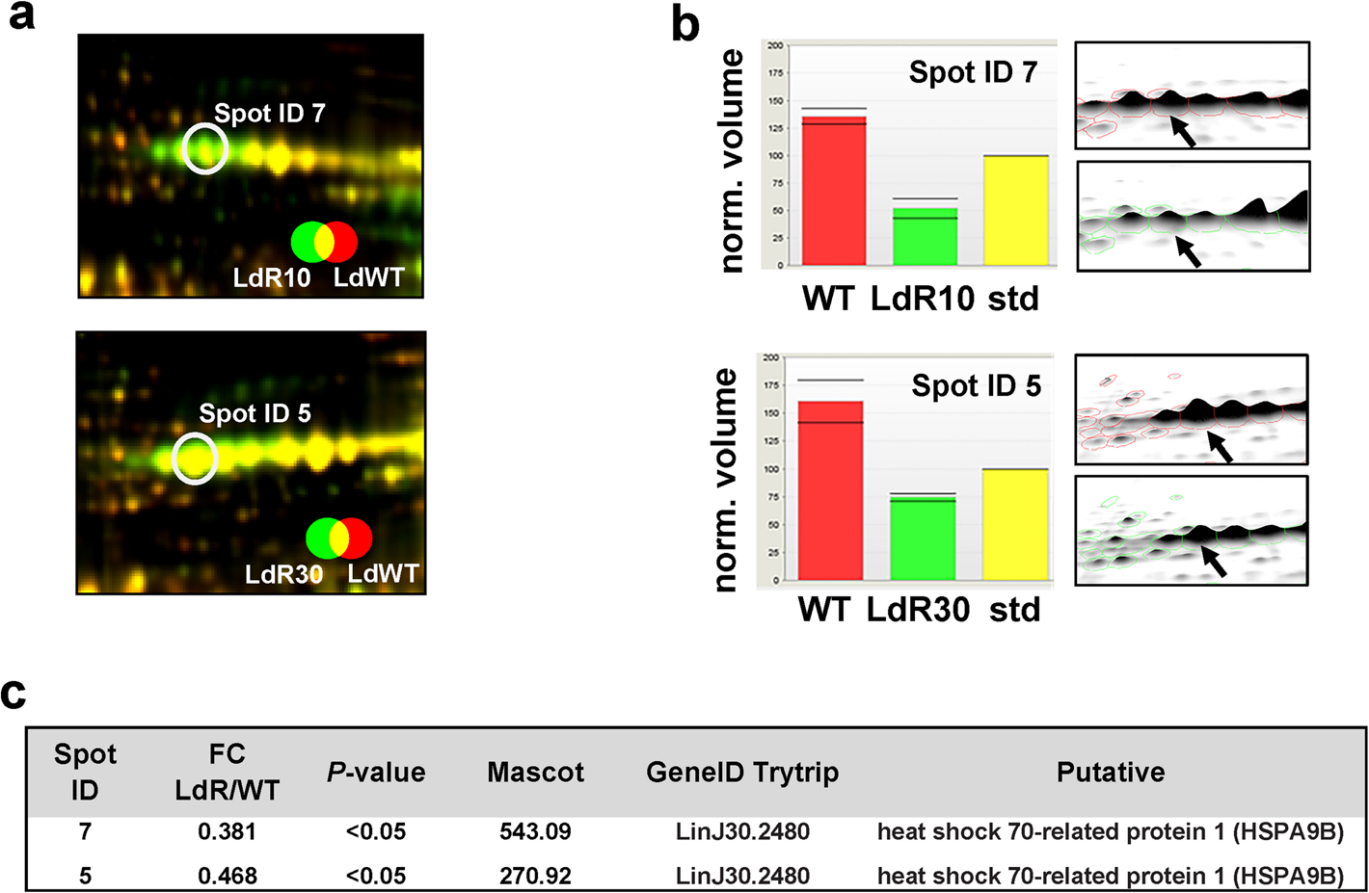
2D-DIGE quantitative analysis of miltefosine-resistant *L. donovani*. **a** Enlarged regions of the 2D-DIGE gels showing Cy3-labeled LdR10 or LdR30 promastigotes and Cy5-labeled WT promastigotes. **b** Spots ID 7 and 5 were underrepresented in LdR10 and LdR30 lines, respectively, and expression profiles with significant statistics is shown along with representative 3D regions of the gels. **c** Spots ID 7 and 5 were excised and later identified by mass spectrometry as LinJ30.2480 (HSPA9B). The table shows Fold Change (FC) of LdR/LdWT ratio, and Mascot and *P*-values for spots ID7 and ID5

In order to identify molecules potentially implicated in HePC resistance, we performed comparative 2D-DIGE of proteins isolated from cultures of *L. donovani* wild type (WT) vs LdR10 (“low” level of resistance) promastigotes and *L. donovani* WT *vs* LdR30 (“high” level of resistance) promastigotes. Whole extracts were obtained, differentially labeled with CyDye fluors as detailed in Methods and separated by 2DE on pH 4–7 IPG immobiline strips and SDS-PAGE. Images were analyzed with Delta2D (Decodon) software. Several proteins were up- or downregulated more than 2-fold with *P*-values < 0.05 (data not shown). Interestingly, Spot ID 7 and Spot ID 5 (Fig. 1a) were underrepresented in both LdR10 and LdR30 (0.381- and 0.468-fold change, respectively, Fig. 1b) and excised to be further identified by MS/MS (Fig. 1c). A representative 2D-DIGE gel image is shown in Additional file 1: Figure S1. The principal components analysis applied on the gels is indicative of the reproducibility between replicates (Additional file 2: Figure S2). Both spots were identified as LinJ30.2480 (Tritryp gene ID). This gene is a putative *heat-shock 70-related protein 1 (mitochondrial precursor)* with a predicted MW of 71.6 kDa (designated as HSPA9B [10]).

**Fig. 2:**
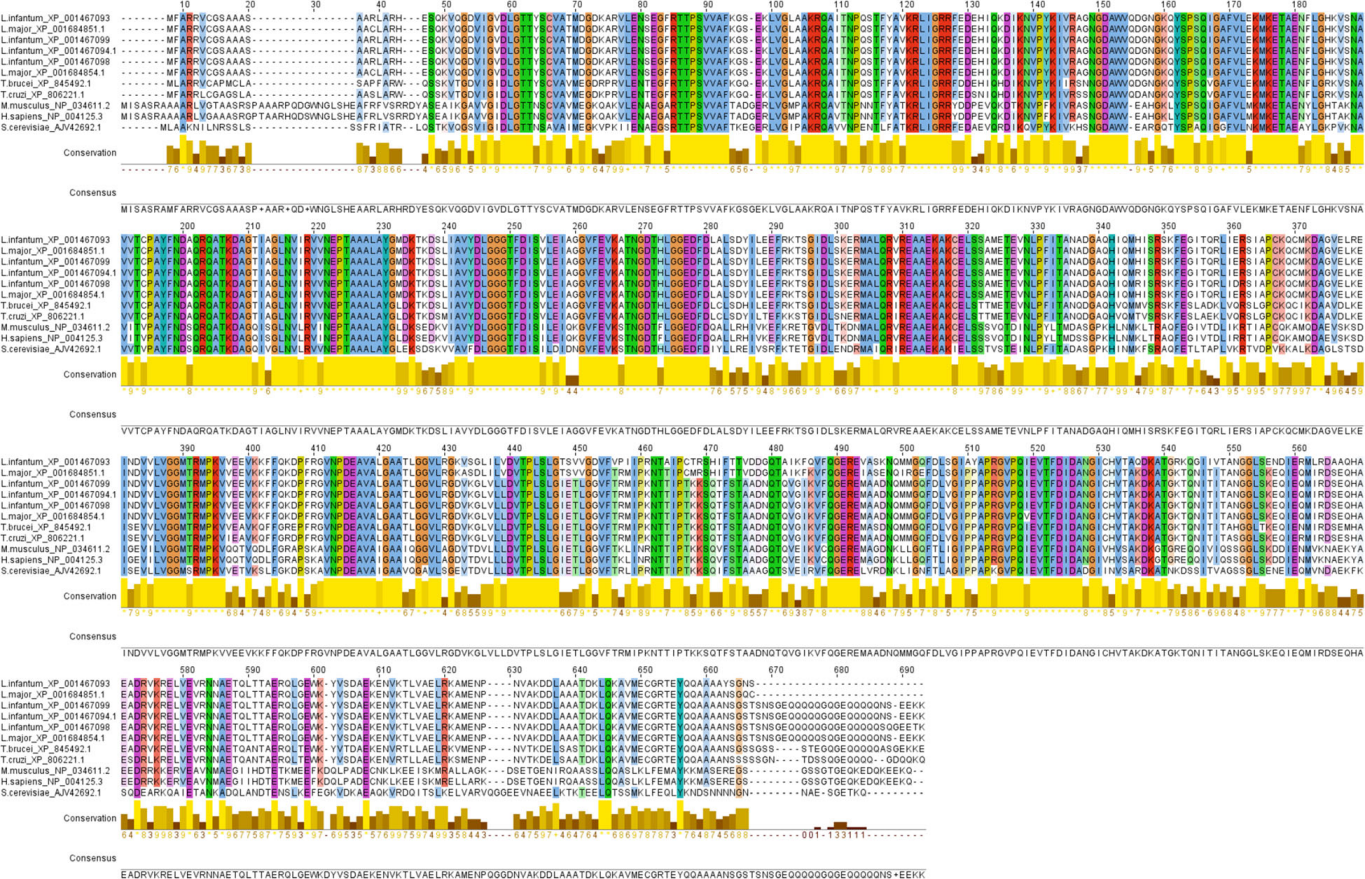
Multiple alignment of *L. donovani* HSPA9B with other species. The amino acid sequence of *Leishmania infantum* (Acc. No. XP 001467093) was aligned with homologs from *Leishmania major* (Acc. Nos. XP 001684851.1 and XP 001684854.1), *Leishmania infantum* (Acc. Nos. XP 001467099, XP 001467094.1 and XP 001467098), *Trypanosoma brucei* (Acc. No. XP 845492.1), *Trypanosoma cruzi* (Acc. No. XP 806221.1), *Mus musculus* (Acc. No. NP 034611.2), *Homo sapiens* (Acc. No. NP 004125.3) and *Saccharomyces cerevisiae* (Acc. No. AJV 42692.1) using Clustal X (v2.1) and edited with Jalview [41]. Conserved (>75%) residues are highlighted and conservation histograms are indicated below each residue

An *in silico* analysis of *L. infantum* database revealed, in total, the existence of four genes, organized in tandem, encoding for HSPA9A-D. Together, they share more than 91% identity (Fig. 2). While the N-terminal segment is conserved, it is the C-terminal end that shows higher divergence. HSPA9B is highly conserved among other *Leishmania* species and related trypanosomatids (*T. cruzi* and *T. brucei*) with identities above 90%. Additionally, HSPA9B shares a 55–60% identity with higher eukaryotes (Fig. 2 and Additional file 3: Figure S3).

To gain a first insight in how HSPA9B expression is associated to HePC resistance we established transgenic parasites overexpressing HSPA9B. This gain-of-function approach allowed us to overcome current limitations ssociated with knock out and knock down strategies, especially because HSPA9B is not a single copy gene.

### Phenotypic characterization of HSPA9B overexpressing parasites

**Fig. 3:**
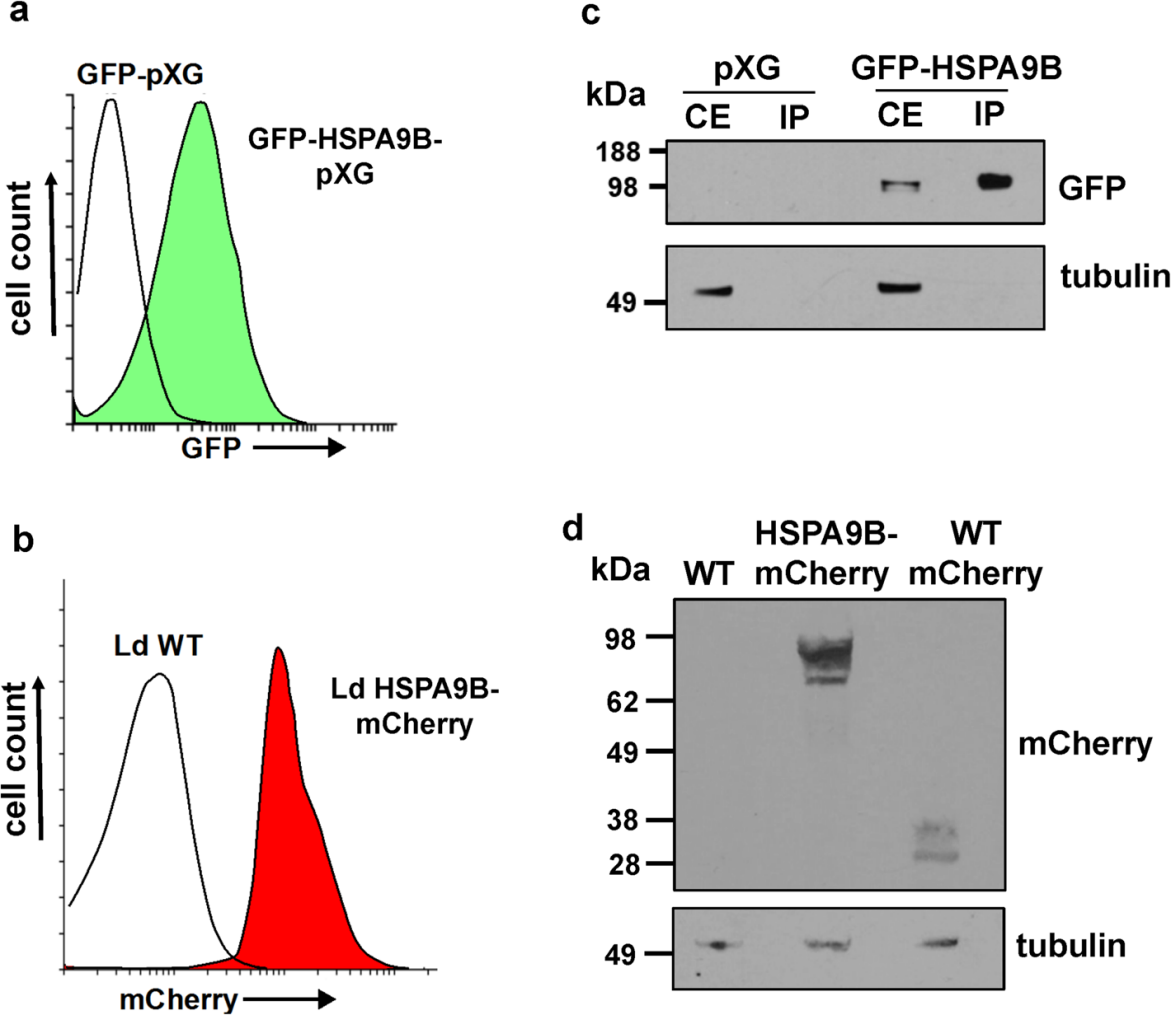
Overexpression of HSPA9B-tagged proteins in *L. donovani* promastigotes. Protein expression in transgenic promastigotes carrying the episomal construction GFP-HSPA9B-pXG (**a**) or the integrative version HSPA9B-mCherry (**b**) were analyzed by flow cytometry and western blot. Histograms indicate fluorescence intensity of controls (*black* line) and transfected parasites (*green* or *red* filled). Total (CE) and immunopurified (IP) protein extracts (**c**) or just total (**d**) extracts obtained from logarithmic cultures were resolved by SDS-PAGE and probed with anti-GFP or anti-mCherry antibodies. Blots were stripped and re-probed with an anti-α-tubulin antibody as a loading control. Molecular weights are indicated in kDa

We followed a gain-of-function approach in order to reveal the influence of HSPA9B in the parasite biology. C-terminal HSPA9B-mCherry (integrative) and N-terminal GFP-HSPA9B (episomal) fusions were generated and protein overexpression was confirmed by western blot and flow cytometry (Fig. 3a, b). Growth curves of the transgenic lines HSPA9B-mCherry, GFP- HSPA9B and their respective controls show that overexpression of HSPA9B does not affect the parasite’s ability to replicate (Additional file 4: Figure S4).

### HSPA9B overexpressing parasites are more sensitive to drug treatment

We investigated the effects of HSPA9B overexpression on parasite response to HePC treatment. The assessment of HePC resistance was measured using the resazurin-based CellTiter-Blue assay described in Methods. Both overexpressing lines generated in this study are more sensitive to HePC treatment, regardless of whether the expression is ectopic or integrative (Table 1). Because we found HSPA9B to be downregulated (Fig. 1) in resistant LdR10 and LdR30 cell lines (Table 1), it seems counterintuitive that the overexpression of HSPA9B confers increased susceptibility to HePC. The HSP70 protein family is structurally characterized by the presence of three conserved domains: at the N-terminus there is a highly conserved 44 kDa ATP-binding/ATPase domain followed by a 13 kDa peptide binding domain containing two antiparallel beta-sheets. At the C-terminal end there is an alpha-helical domain of *c.*10 kDa whose sequence is less conserved between distantly related organisms (Fig. 2). To ascertain the potential and specific role of HSPA9B in HePC resistance, we decided to test the effect of dominant-negative mutants on drug susceptibility. The constructs HSPA9B-mCherry (G220D, G221D, denoted as single mutations) and HSPA9B-mCherry (G220D, G221D, F436V, denoted as double mutations) were designed based on previous studies where the relevance of specific amino acids in the ATP-binding domain and peptide binding domain were assessed [15–18]. Following transfection, we measured HePC sensitivity in transgenic lines (HSPA9B-mCherry) carrying single (SM) or double mutations (DM) (Table 1), as described above. The EC_50_ values of lines carrying these mutations suggest that the expression of mutant polypeptides in a WT background significantly alters HSPA9B activity. Furthermore, both single and double mutations are able to revert the HePC-sensitive phenotype of HSPA9B-mCherry transgenic parasites to WT values (Table 1). Overall these data suggest a specific and important role of HSPA9B in HePC resistance.

**Table 1:** Miltefosine susceptibility of *L. donovani* populations determined by EC_50_ assays. Upon analysis, parasite lines show increasing degrees of resistance to HePC as selection pressure increases (denoted as LdR10 and LdR30, respectively). Transgenic lines over-expressing HSPA9B show increased sensitivity to drug treatment as compared to their controls. Sensitivity reverts back to WT like values upon generating mutations in the ATP binding domain (GxD, GxD, referred to as SM) or both the ATP and peptide binding domains (GxD, GxD, FxV, referred to as DM)

EC_50_ (mean ± SD for at least 3 replicates)								
	HePC resistant lines^a^		Transgenic lines				HSPA9B-mCherry mutants	
	LdR10	LdR30	pXG	GFP-HSPA9B	WT	HSPA9B-mCherry	SM	DM
HePC	38.99 ± 5.06	94.04 ± 16.82	8.14 ± 0.45	5.76 ± 0.53	7.04 ± 0.36	3.92 ± 0.23	6.7 ± 0.27	6.95 ± 0.61

^a^These EC_50_ values were previously determined and published by our group [9]

Although *L. donovani* HePC-resistant lines (LdR30) did not show cross-resistance against the reference compounds paromomycin, amphotericin B, antimony (III) and pentamidine isethionate [9], we decided to investigate the possibility of any conferred resistance to the alternative antileishmanial treatments by measuring EC_50_ values in HSPA9B-mCherry overexpressing lines. Unexpectedly, we found that these cells are significantly more resistant to amphotericin B, antimony (III) and pentamidine isethionate when compared to the WT control (Additional file 5: Table S1). Further studies will be necessary to understand the role of HSPA9B in resistance to these drugs.

### Intracellular accumulation of HePC in transgenic parasites

**Fig. 4:**
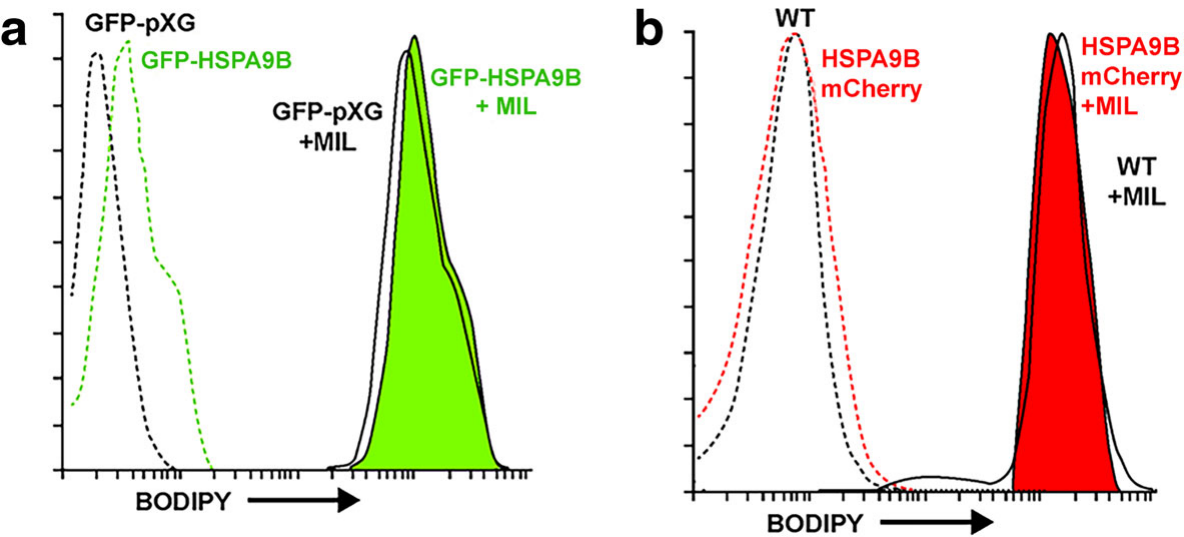
Miltefosine incorporation in transgenic *L. donovani* parasites. Cells were incubated with 5 μM MIL-BODIPY and subsequently washed with 4% BSA in HPMI buffer (w/v) and analyzed by flow cytometry. **a** MIL-BODIPY uptake in GFP-HSPA9B-pXG (*green* filled line) and mock parasites (*black* line). **b** MIL-BODIPY accumulation in HSPA9B-mCherry (*red* filled line) and WT parasites (*black* line). Culture auto-fluorescence is indicated as dotted lines. These are representative histograms of at least three independent experiments

Previous reports [6, 7, 9, 31–36] have linked reduced HePC sensitivity to impaired drug incorporation or increased drug extrusion. To assess the ability of HSPA9B overexpressing transgenic parasites to accumulate HePC, we monitored the uptake of HePC-BODIPY by flow cytometry. Under our experimental conditions, we do not detect significant differences in drug accumulation, as judged by the similar fluorescence levels (histograms) of transgenic HSPA9B cultures and their respective controls (Fig. 4). These data suggest that the augmented HePC susceptibility we observe in the HSPA9B overexpressing cultures is not likely caused by an increased uptake of the HePC fluorescent analog.

### Subcellular localization of HSPA9B

**Fig. 5:**
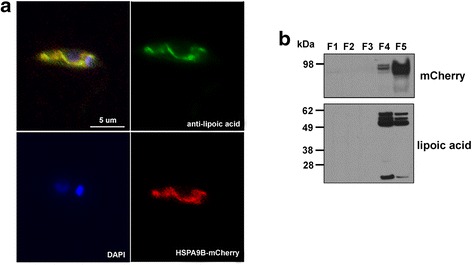
Subcellular localization of HSPA9B. **a** Immunofluorescence microscopy of HSPA9B-mCherry promastigotes (*red*) using a primary polyclonal antibody against lipoic acid and a FITC-conjugated anti-rabbit (*green*) as a secondary antibody. DNA was stained with NucBlue fixed cell stain (DAPI, blue). *Scale-bar*: 5 μm. Images were obtained with a Nikon 90/NikonAZ 100 microscope. **b** Western blot of subcellular fractions obtained by digitonin fractioning of HSPA9B-mCherry parasites. Samples were separated by SDS-PAGE, transferred on to a PVDF membrane and assayed with anti-mCherry antibody. The membrane was stripped and re-probed with a polyclonal anti-lipoamide antibody. Molecular weights are indicated in kDa. Fractions (F) 1 and 2 contain cytosolic proteins, mitochondrial proteins are mostly recovered in fractions (F) 3 and 4; fraction (F) 5 is enriched in cytoskeleton and cytoskeleton-associated proteins

To ascertain the subcellular localization of HSPA9B, we performed immunofluorescence microscopy of *L. donovani* HSPA9B-mCherry promastigotes (Fig. 5a). The fluorescence pattern of HSPA9B mimics traditional mitochondrial localization as evidenced by the yellowish fluorescence, which corresponds to co-localization with the polyclonal anti-lipoic acid. This antibody recognizes lipoic acid and lipoic acid covalently attached to proteins via a lipoamide bond, a cofactor associated to E2 subunits of mitochondrial protein complexes like pyruvate-dehydrogenase, alpha-ketoglutarate dehydrogenase and the branched-chain alpha-keto acid. Additional evidence of HSPA9B mitochondrial localization was obtained from subcellular fractioning of *Leishmania* cultures using increasing digitonin concentrations, allowing progressive membrane permeabilization. At low digitonin concentrations, only the plasma membrane becomes permeable, however higher concentrations are able to sequentially extract organelle components. The selective permeabilization was carried out according to the Foucher et al. fractioning method [29]. According to this protocol, cytosolic proteins are mainly found in fractions 1 and 2, mitochondrial proteins are mostly recovered in fractions 3 and 4 while fraction 5 is mainly enriched in cytoskeleton and cytoskeleton-associated proteins. Fractions were separated by SDS-PAGE, underwent western blotting and were further incubated with anti-mCherry and anti-lipoic acid antibodies. The 98 kDa HSPA9B-mCherry fusion protein, recognized by the mCherry antibody, is detected in fractions 4 and 5. A similar result is obtained with anti-lipoic acid (Fig. 5b). Together, our results confirm the mitochondrial localization of HSPA9B.

### Effect of environmental stress on transgenic HSPA9B parasites

*Leishmania* spp*.* are digenetic pathogens where temperature shift takes place as part of their life-cycle. Phases of low temperatures in the insect vector and higher temperatures in the mammalian host are accompanied by a strong induction of heat-shock proteins [14, 37]. Increased expression of HSPs protect the cell by stabilizing unfolded proteins, giving the cell time to repair or re-synthesize damaged proteins during heat-shock transitions.

**Fig. 6:**
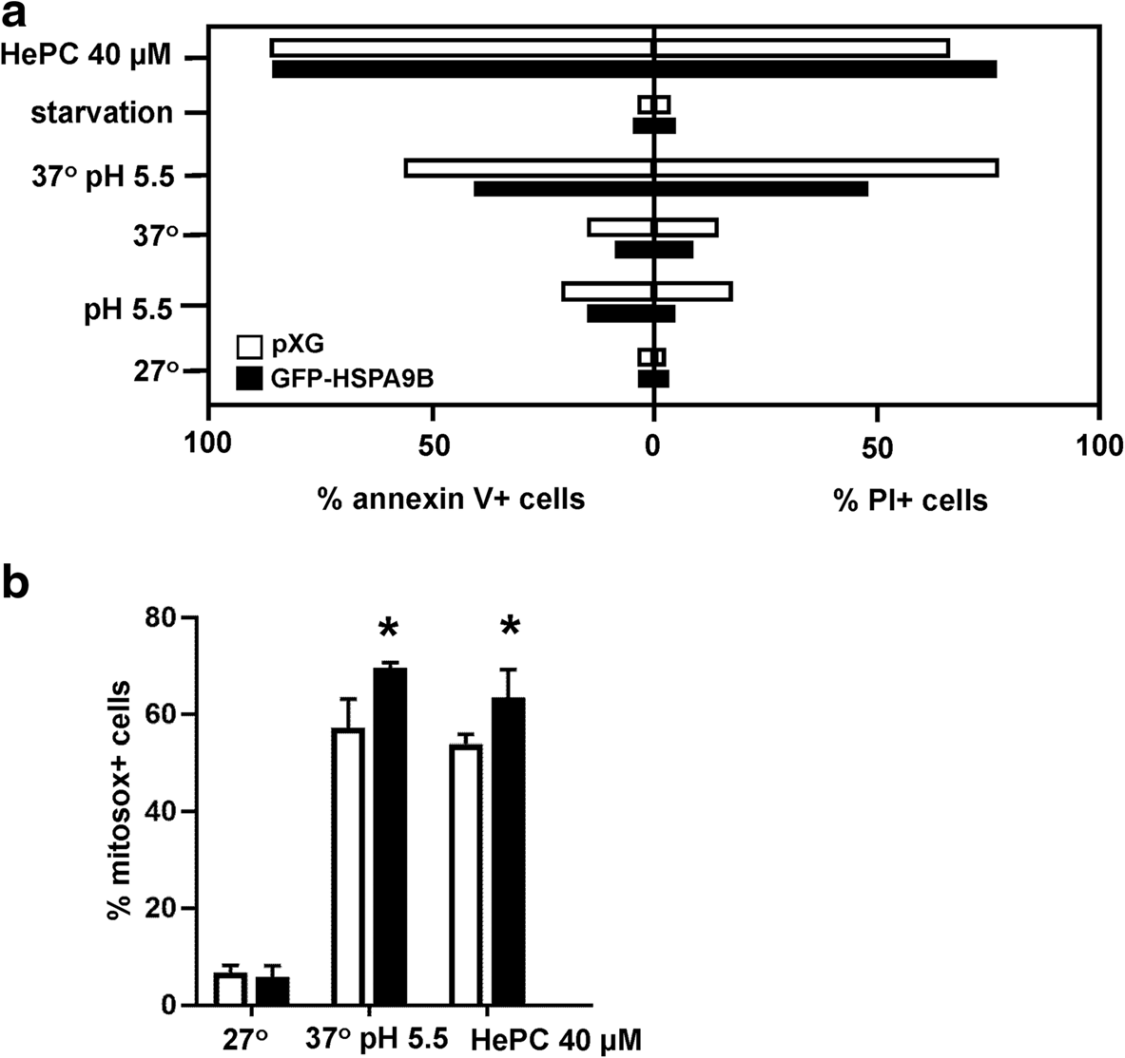
Environmentally-induced stress response and ROS generation in *L. donovani* overexpressing HSPA9B. **a** Promastigotes were exposed to low pH (pH = 5.5), heat-shock (37 °C), heat-shock (37 °C) plus low pH (pH = 5.5), starvation and HePC treatment. Control cells were incubated at 27 °C. The percentage (%) of early apoptotic-AnnexinV positive cells and late apoptotic-PI positive cells was measured by flow cytometry. These are representative histogram bars of at least two independent experiments. **b** Production of intracellular ROS under low pH (pH = 5.5), heat-shock (37 °C) plus low pH (pH = 5.5) and HePC treatment was measured by flow cytometry using MitoSOX Red. Data are the means ± standard deviations (SD) of three independent experiments. *P < 0.05

We analyzed transgenic *L. donovani* promastigotes overexpressing HSPA9B, both integrated or episomally, in order to investigate how HSPA9B may affect the ability of cells to cope with stressful environments. Parasites were exposed to different stress conditions: low pH (pH = 5.5), heat-shock (37 °C), heat-hock (37 °C) plus low pH (pH = 5.5), starvation and HePC treatment during 4 and 18 h. Cell health was monitored by measuring early (PS exposure) and late (PI permeability) apoptotic indicators. Analyses were performed in a Beckman Coulter FC500 Flow Cytometer and FSC *vs* SSC plots were used for gating cells and for identifying changes in the scatter properties of the cells. After 4 h, both exposed and non-exposed cultures are viable and non-apoptotic. Substantial changes are only detected after 18 h of treatment (Fig. 6a). For all lines analyzed, low pH, high temperature and a combination of both significantly increase the number of stressed cells, evidenced by the augmented percentage of early and late apoptotic parasites when compared to normal growth conditions (27 °C, pH = 7.2) (Fig. 6a). Interestingly, transgenic HSPA9B parasites elicit a moderate stress response, with *c.*40% of cells positive for PS and *c.*50% positive for PI (Fig. 6a). Conversely, control lines display *c.*60% and 80% positive cells for PS and PI, respectively, suggesting that HSPA9B overexpression is able to protect cells from taxing environmental changes.

Mitochondria superoxide accumulation was also tested in the same conditions previously described. Superoxide anion levels were followed with MitoSox in a Beckman Coulter FC500 Flow Cytometer. While pH media reduction alone does not modify the radical production of superoxide in transgenic lines when compared to controls, temperature shift along with low pH or incubation with 40 μM HePC increases significantly superoxide accumulation in overexpressing cultures (Fig. 6b), suggesting that these parasites are more susceptible to oxidative stress damage. To further study this possibility we performed comparative proteomics experiments of mitochondrial protein enriched extracts.

### 2D-DIGE of membrane-associated proteins from HSPA9B transgenic lines

**Fig. 7:**
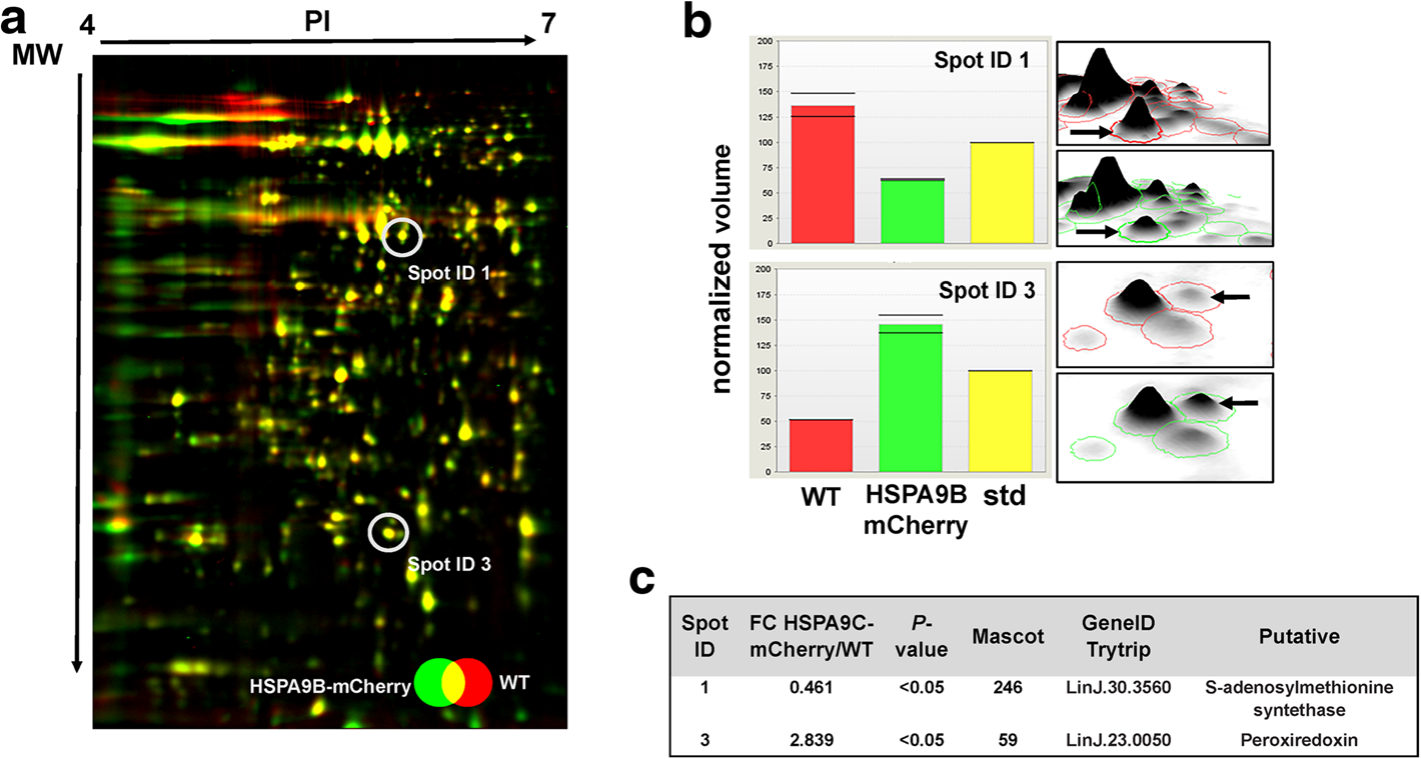
2D-DIGE quantitative analysis of the membrane-associated proteome of HSPA9B-mCherry transgenic *L. donovani*. **a** Protein extracts were enriched for mitochondrial membrane and lumen components using the Qproteome Cell Compartment Kit. A representative gel image is shown. **b** Spots ID 1 and 3 are under- and overrepresented, respectively, in the HSPA9B-mCherry line and expression profiles with significant statistics is shown along with representative 3D regions of the gels. **c** After image analysis, spots were excised and identified by mass spectrometry. The table shows the Fold Change (FC) HSPA9B-mCherry/WT ratio, as well as Mascot and *P*-values for spots ID1 and ID3

Protein extracts enriched in mitochondrial membrane and lumen components were obtained by fractionation with the Qproteome Cell Compartment Kit. After labelling, extracts from WT and HSPA9B-mCherry transgenic lines were subjected to 2D-DIGE analysis as described in Methods (Fig. 7a). Spot ID 1 (0.461-fold change; *P* = 0.019) and Spot ID 3 (2.839-fold change; *P* = 0.0032) (Fig. 7b) were identified as a S-adenosylmethionine synthetase –S-AdoMetS- (LinJ.30.3560) and peroxiredoxin –mTXPNx- (LinJ.23.0050), respectively (Fig. 7c). The principal components analysis applied on the gels is indicative of the reproducibility between replicates (Additional file 6: Figure S5). These two enzymes have been implicated in redox metabolism in trypanosomatids [38–40]. Specifically, S-AdoMetS is responsible for adenosylmethionine synthesis, a key compound in the trypanothione biosynthetic pathway [38], and has been recently linked to methotrexate resistance [39, 41]. mTXPNx, however, is a mitochondrial protein and its expression has been associated, in *L. donovani,* with reduced susceptibility to programed cell death (PCD) [40].

### Phenotypic characterization of axenic amastigotes overexpressing HSPA9B

**Fig. 8:**
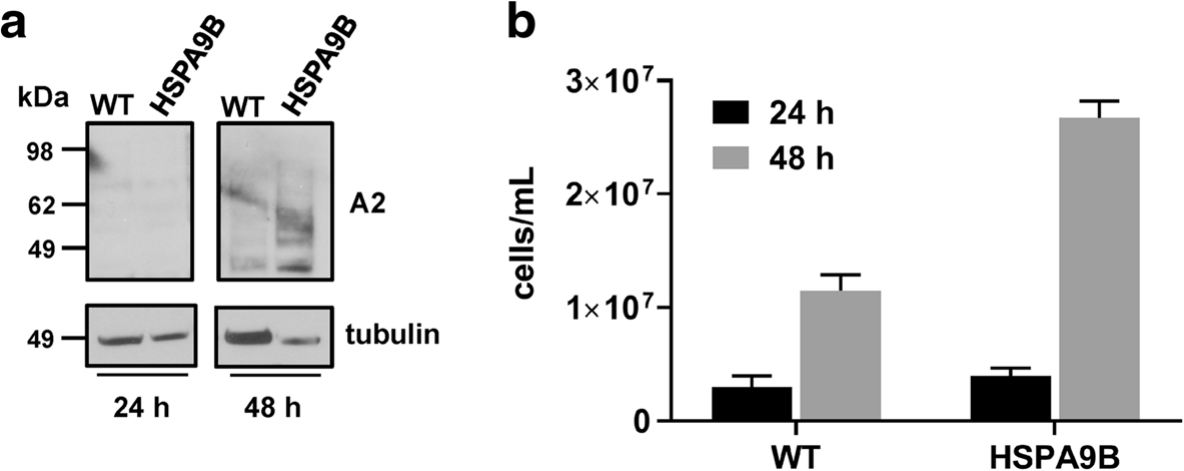
Effect of HSPA9B overexpression on differentiation. **a** *L. donovani* WT and transgenic promastigotes (HSPA9B-mCherry) were inoculated in low pH (pH = 5.5) medium and 37 °C to trigger differentiation to axenic amastigotes. Cells were collected 24 and 48 h post-induction of differentiation and either lysed for western blotting with axenic amastigote A2 marker and anti-tubulin as a loading control or counted using a Neubauer chamber. **b** The molecular weight of standard proteins is indicated in kDa and the mean ± standard deviation (SD) of triplicate determinations are shown, respectively

Our results suggest that HSPA9B overexpression protects parasites against changes in stressful environmental conditions such as low pH and high temperature. Because *Leishmania* parasites face similar environmental conditions upon infection of host cells and differentiation into amastigotes we sought to determine the effects of HSPA9B overexpression on differentiation into axenic amastigotes and further, on proliferation. *L. donovani* transgenic (HSPA9B-mCherry) and WT promastigotes from a late-log culture were inoculated in low pH medium and subjected to 37 °C and 5% CO_2_ incubation to trigger differentiation into axenic amastigote. Cells were lysed 24 and 48 h post-differentiation, and a western blot was performed with axenic amastigote marker A2 and anti-tubulin as a loading control (Fig. 8a). Additionally, the number of fully differentiated cells was determined at each time point (Fig. 8b). Both WT and HSPA9B-mCherry cell lines are able to fully differentiate into axenic amastigotes within 48 h, as judged by the expression of A2. It is important to note that the transgenic line displays a higher number of amastigotes, suggesting once again that the overexpression of HSPA9B may confer *Leishmania* an advantage to cope with the harsh environment encountered during differentiation.

## Discussion

We have previously applied next-generation sequencing (NGS) technologies to better comprehend the mechanisms of HePC resistance in *L. donovani* [9]. Even though observations of clinical resistance to HePC are scarce, its long half-life (approximately 120 h) and low therapeutic window make the emergence of resistance on a larger scale a very likely process. Until fully characterized resistant field isolates become available, experimental selection of HePC resistance in the laboratory may offer insight into potential mechanisms of resistance and contribute to design strategies to prevent emergence and spread of resistance.

An interesting feature of the *Leishmania* (and other trypanosomatids) genome is the unusual nature of transcription and RNA processing. Protein coding genes are organized into long gene clusters [42, 43], and the resulting polycistronic RNAs are post-transcriptionally processed into mature mRNAs by concomitant trans-splicing and polyadenylation. In addition, trypanosomatids appear to have few of the regulatory transcription factors found in higher eukaryotes [43] and transcription initiates at fewer than 200 sites in the entire genome [44, 45]. Thus, these organisms largely lack the transcriptional regulation of gene expression characteristic of other eukaryotes [45] and, as a consequence, proteomic studies often render a more useful view of the biology of the parasite. We performed quantitative in-gel proteomics comparing control WT and HePC-resistant LdR10.1 and LdR30.1 *L. donovani* promastigotes (Fig. 1) and identified HSPA9B, a mitochondrial HSP70, downregulated in both resistant lines.

Proteins of the HSP70 family are central components of many fundamental cellular processes, including the folding and assembly of newly synthesized proteins and refolding of misfolded and aggregated proteins. Members of the HSP70 family localized in the mitochondria, such as HSPA9B, are most similar to the bacterial DnaK [13]. Moreover, in most organisms, ER and mitochondrial HSP70s are constituted by a sole protein, however an interrogation of the *L. donovani* genome revealed four HSPA9 genes (A-D) with minor sequence differences suggesting divergent evolution of this protein subfamily [10]. A previous report from 2008 demonstrated the existence of two distinct HSPA9B homologs in *L. chagasi* fortuitously isolated from an immunoscreening of a *L. chagasi* cDNA expression library [14] showing the same sequence arrangement.

Besides their role in thermotolerance, it has been shown that HSPs also play a role in resistance to some antileishmanial compounds, particularly antimonials. For instance, cosmids recovered from SbIII resistant promastigotes contained two copies of cytoplasmic HSP70 and one copy of HSC70 [46]. Moreover, the expression of HSP70 was augmented when cells were grown at 2× the SbIII EC_50_, suggesting a link of HSP70 to antimony response and resistance. It was also observed that transfection of HSP70 does not confer resistance to SbIII directly.

We seemingly uncovered a link between HePC resistance and HSPA9B expression in our 2D-DIGE experiment and generated an overexpression system to further corroborate this connection. Our gain-of-function approach demonstrates that transgenic lines overexpressing HSPA9B are more susceptibility to HePC (Table 1). This phenotype is independent of the method employed, i.e. integrative (HSPA9B-mCherry) or episomal (GFP-HSPA9B). Furthermore, our data suggest that the increased sensitivity to HePC is specific to HSPA9B overexpression since dominant negative mutant lines (HSPA9B -mCherry (GxD, GxD) and HSPA9B -mCherry (GxD, GxD, FxV)) are able to restore HePC susceptibility to WT values (Table 1), emphasizing the importance of the ATP-binding and peptide binding domains for the proper function of HSPA9B. HePC internalization remains the main determinant of drug susceptibility and it correlates with the levels of the HePC translocation complex [36]. Remarkably, we found that the augmented susceptibility of HSPA9B transgenic lines to HePC does not correlate with an increased HePC uptake (Fig. 4). Although we did not measure directly the expression levels of LdMT and LdRos3, our data indirectly suggest that HePC is internalized at a similar rate based on the accumulation of the HePC fluorescent analog, underscoring the multi-factorial mechanism of HePC resistance in *Leishmania* spp.

As mentioned above, HSPs have been previously implicated in drug resistance with the analysis of antimony-resistant clinical isolates of *L. donovani*. In one analysis, both cytoplasmic and mitochondrial forms of HSP70 were upregulated (up to 5-fold) in resistant isolates [47]. Authors concluded that the most plausible explanation is that HSP70 may protect *Leishmania* from drug-induced programmed cell death (PCD). Antimonials kill cells via a process reminiscent of PCD, and thus HSP70 may have an anti-apoptotic effect. A similar mechanism has been proposed for HePC, in which cells are killed by a process leading to PCD, including cell shrinkage, DNA fragmentation and changes in membrane composition. The downregulation of HSPA9B in HePC-resistant *L. donovani* may have an indirect anti-apoptotic effect and we speculate that HSP9C cooperates closely with other chaperones in the matrix compartment, e.g. HSP60, altering the network of mitochondrial chaperones and ultimately protecting mitochondrial proteins from misfolding due to HePC stress.

In order to better understand the role of HSPA9B in the biology of the parasite we subjected *L. donovani* promastigotes overexpressing HSPA9B to different stressful environments. After 18 h of treatment (a combination of low pH and high temperature) transgenic lines display a lower percentage of early (PS exposure) and late (PI) apoptotic cells (Fig. 6a) suggesting that HSPA9B is capable of protecting cells from stressful environmental conditions. This phenotype was further corroborated in axenic amastigotes transfected with an episomal copy of HSPA9B. These transgenic amastigotes differentiate and proliferate faster than control cells (Fig. 8) confirming the potential protective role of HSPA9B upon environmental stress. These data highlight a fascinating functional dichotomy for HSPA9B: its downregulation in HePC resistant lines may protect cells from drug-induced PCD, however its overexpression, unexpectedly, does not increase the sensitivity of *Leishmania* to environmental stress and, on the contrary, we observe a protective role under low pH and high temperature.

Several studies have pinpointed mitochondrial-dependent apoptosis and generation of ROS as major effectors in the mechanism of action of HePC [48, 49]. We measured superoxide accumulation with the fluorescent dye MitoSox (indicative of ROS levels in the mitochondria) under low pH and high temperature, and showed that transgenic parasites overexpressing HSPA9B are more susceptible to oxidative stress damage (Fig. 6b). This result may support one of the potential functions of HSPA9B: the underexpression of HSPA9B in the HePC resistant lines generated in our laboratory partially protects the cells against drug-induced oxidative stress. Furthermore, it is well known that *L. donovani* promastigotes resistant to HePC usually exhibit different metabolic adaptations leading to protection against oxidative stress [49]. In this regard we performed 2D-DIGE analysis of enriched mitochondrial proteins from HSPA9B overexpressing promastigotes and control cells (Fig. 7). S-adenosylmethionine syntethase is downregulated (0.461-fold change) in transgenic lines and from this we may infer that the levels of its substrate, AdoMet, part of the trans-sulfuration pathway, may also be affected. Changes in AdoMet production have been identified in drug resistant *Leishmania* and a correlation with oxidative stress has been established [49]. The second protein identified in our comparative proteomics analysis (Fig. 7) is a mitochondrial peroxiredoxin, mTXNPx, upregulated in transgenic lines. Remarkably, Teixeira et al. have shown that mTXNPx functions as a temperature-sensitive cochaperone in *L. infantum* [50]*.* In this study it was demonstrated that mTXNPx prevents protein aggregation in *Leishmania* promastigotes under temperature of 37 °C. Briefly, the change in temperature activates the chaperone function of mTXNPx causing it to bind to client unfolded proteins to prevent them from forming cytotoxic aggregates. These client proteins can then be reactivated by members of the HSP70 system. We hypothesize that HSP9AC overexpression leads to an overexpression of mTXNPx and as a result cells are more protected from environmental stress that includes changes in temperature and pH. It would be interesting in future studies to reveal the potential interaction of HSPA9B and mTXNPx, along with other members of the mitochondrial chaperone network.

## Conclusions

Experimental resistance to HePC is not yet fully understood. We have used in this study quantitative in-gel proteomics to compare HePC-resistant and sensitive *L. donovani* lines previously generated and have identified a mitochondrial HSPA9B. The role of HSPA9B in HePC resistance is evidenced by a gain-of-function approach and mutagenesis analysis. Interestingly, we have also revealed some of the fundamental roles of HSPA9B in the biology of *Leishmania* such as its implication in environmental and oxidative stress. Overall, our findings are relevant for current and future antileishmanial chemotherapy strategies.

## Additional files

Additional file 1: Figure S1. Representative 2D-DIGE images of LdWT *vs* LdR10 (left panel) and LdWT *vs* LdR30 (right panel). Samples were differentially labeled with fluorescent CyDyes, separated on a 2D-DIGE gel using 13 cm pH 4–7 strips in the first dimension and 12% SDS PAGE gels in the second dimension, and revealed with a Typhoon fluorescent scanner. (PDF 985 kb)
Additional file 2: Figure S2. Principal components analysis (PCA) of LdWT *vs* LdR10/LdR30 2D-DIGE. Statistical analysis shows the “good” clustering of the biological replicates for each experiment: (a) WT *vs* LdR10 and (b) WT *vs* LdR30). (PDF 64 kb)
Additional file 3: Figure S3. Bioinformatic analysis of *L. infantum* (LinJ.30.2480). The relationship of *L. infantum* HSPA9B to other HSPA9B homologs of *L. infantum*, *L. major*, *T. brucei*, *T. cruzi*, *S. cerevisiae*, *M. musculus* and *H. sapiens* was analyzed by multiple alignment and cluster analysis using Clustal X. Alignment was fed into MEGA 6.0 software and a Neighbor-Joining tree was computed with 500 bootstrap replicates. Numbers on the notes indicate bootstrap support. (PDF 10 kb)
Additional file 4: Figure S4. Growth curves of *L. donovani* transgenic lines. Parasite concentration of (a) GFP-pXG mock and GFP-HSPA9B-pXG and (b) WT and HSPA9B-mCherry promastigote cultures was measured microscopically by counting cells in a Neubauer chamber daily for 5 days. The mean ± SD of triplicate determinations are shown. (PDF 36 kb)
Additional file 5: Table S1. Cross-resistance to reference compounds. HSPA9B-mCherry overexpressing lines are significantly more resistant to amphotericin B, antimony (III) and pentamidine isethionate when compared to WT cultures. All the cultures were analyzed in triplicate and data is indicated as mean ± SD of three independent experiments. (PDF 40 kb)
Additional file 6: Figure S5. Principal components analysis (PCA) of WT *vs* HSPA9B-mCherry 2D-DIGE. Statistical analysis shows the “good” clustering of the biological replicates for each sample. (PDF 41 kb)

## Abbreviations

2D-DIGE: Two-dimensional fluorescence difference gel electrophoresis
ATP: Adenosine triphosphate
BODIPY: Boron-dipyrromethene
BSA: Bovine serum albumin
DAPI: 4′,6-diamidino-2-phenylindole
DTT: Dithiothreitol
ER: Endoplasmic reticulum
FCS: Fetal calf serum
FSC: Forward scatter
HePC: Hexadecyl-phosphatidylcholine
HRP: Horseradish peroxidase
HSP: Heat-shock protein
HYGB: Hygromycin
IPG: Immobilized pH gradient
MS: Mass spectrometry
MT: Miltefosine transporter
PCD: Programed cell death
SDS: Sodium dodecyl sulfate
SSC: Side scatter
UV: Ultraviolet
VL: Visceral leishmaniasis
WT: Wild type
