# Supplementary figures and images for: A mitochondrial HSP70 (HSPA9B) is linked to miltefosine resistance and stress response in Leishmania donovani
Source: Parasit Vectors. 2016 Dec 1;9:621. doi: 10.1186/s13071-016-1904-8 (PMC5133764; doi:10.1186/s13071-016-1904-8)

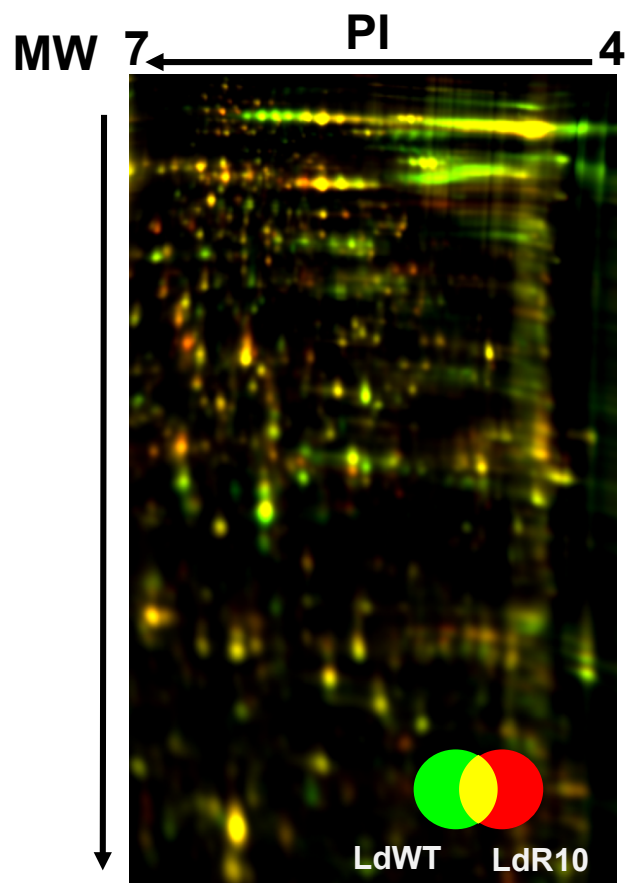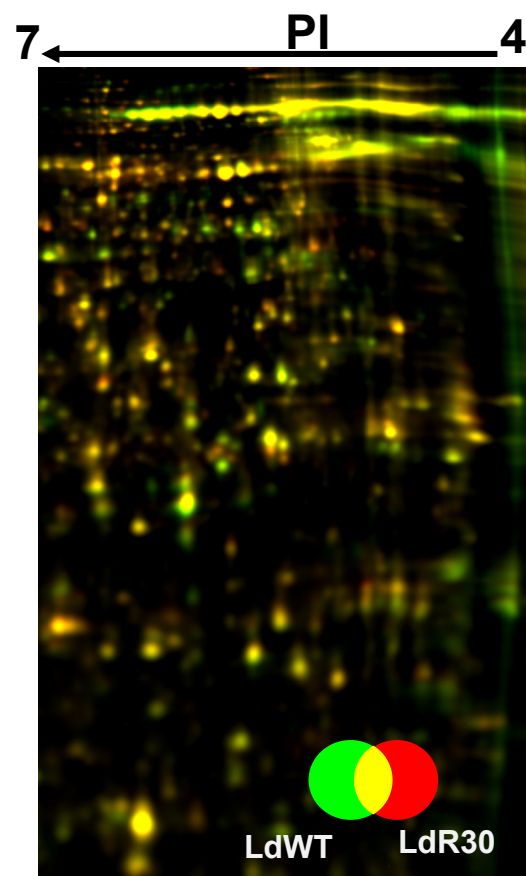

Supplement: Additional file 1: Figure S1. — Representative 2D-DIGE images of LdWT vs LdR10 (left panel) and LdWT vs LdR30 (right panel). Samples were differentially labeled with fluorescent CyDyes, separated on a 2D-DIGE gel using 13 cm pH 4–7 strips in the first dimension and 12% SDS PAGE gels in the second dimension, and revealed with a Typhoon fluorescent scanner. (PDF 985 kb) [file 13071_2016_1904_MOESM1_ESM.pdf]

a

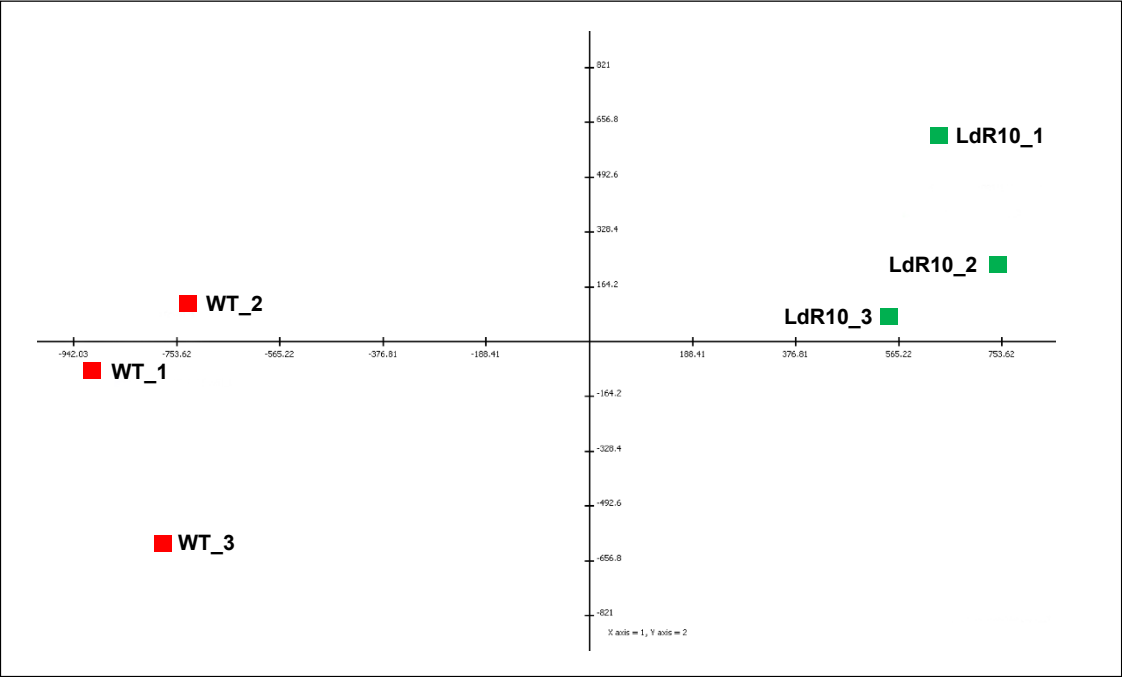

b

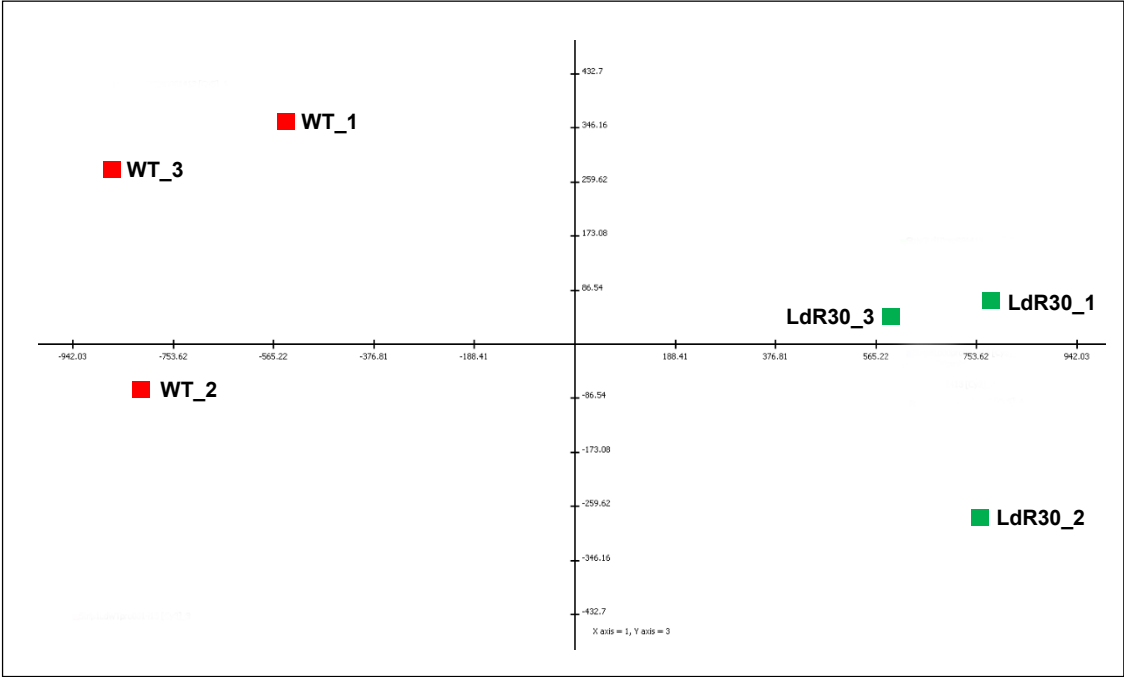

Supplement: Additional file 2: Figure S2. — Principal components analysis (PCA) of LdWT vs LdR10/LdR30 2D-DIGE. Statistical analysis shows the “good” clustering of the biological replicates for each experiment: (a) WT vs LdR10 and (b) WT vs LdR30). (PDF 64 kb) [file 13071_2016_1904_MOESM2_ESM.pdf]

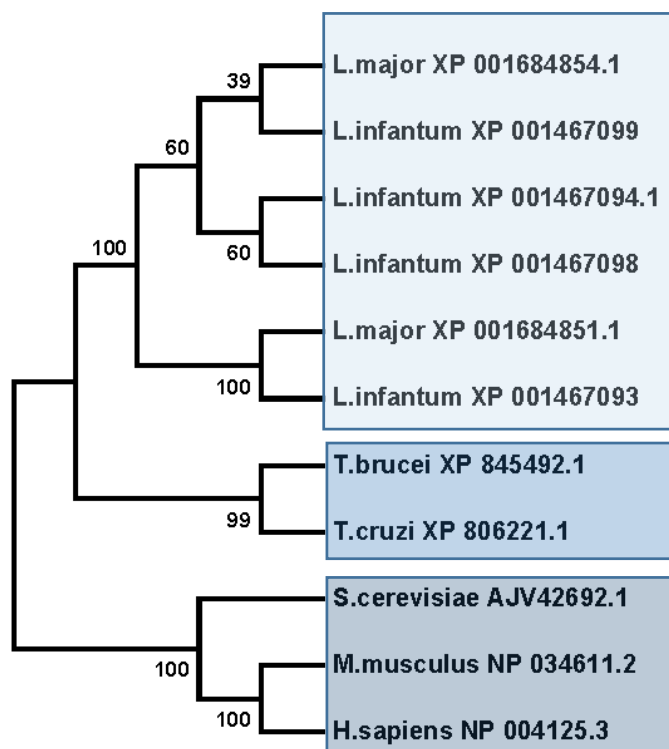

Supplement: Additional file 3: Figure S3. — Bioinformatic analysis of L. infantum (LinJ.30.2480). The relationship of L. infantum HSPA9B to other HSPA9B homologs of L. infantum, L. major, T. brucei, T. cruzi, S. cerevisiae, M. musculus and H. sapiens was analyzed by multiple alignment and cluster analysis using Clustal X. Alignment was fed into MEGA 6.0 software and a Neighbor-Joining tree was computed with 500 bootstrap replicates. Numbers on the notes indicate bootstrap support. (PDF 10 kb) [file 13071_2016_1904_MOESM3_ESM.pdf]

**a**

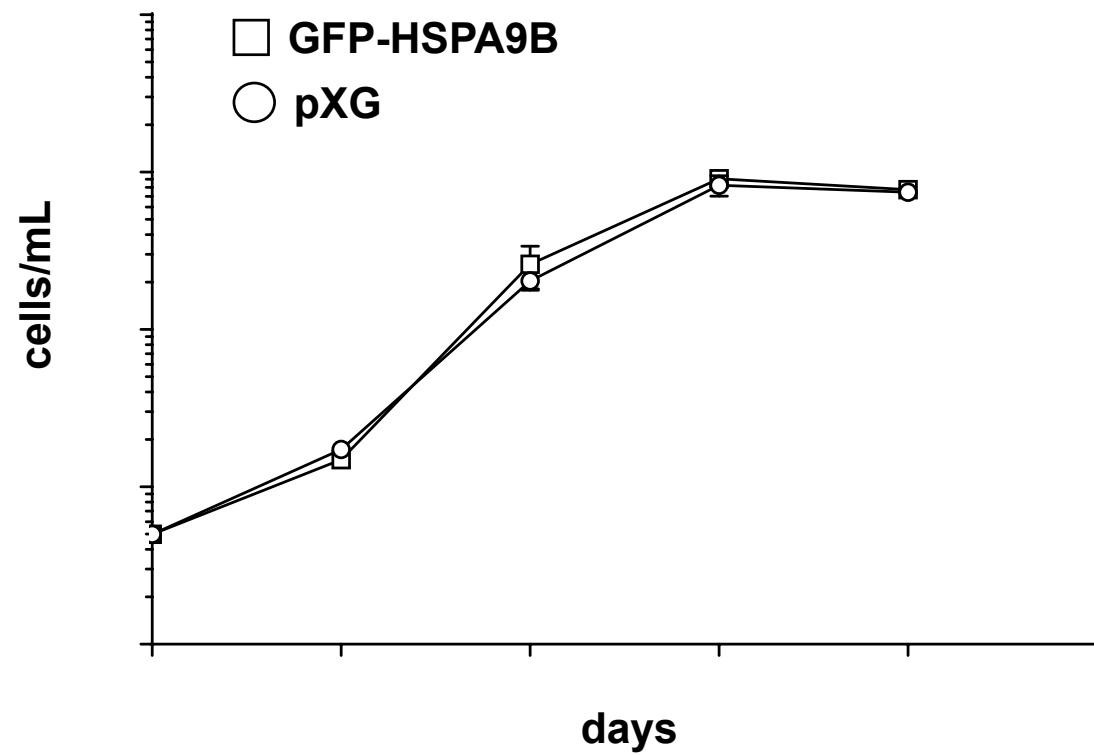

**b**

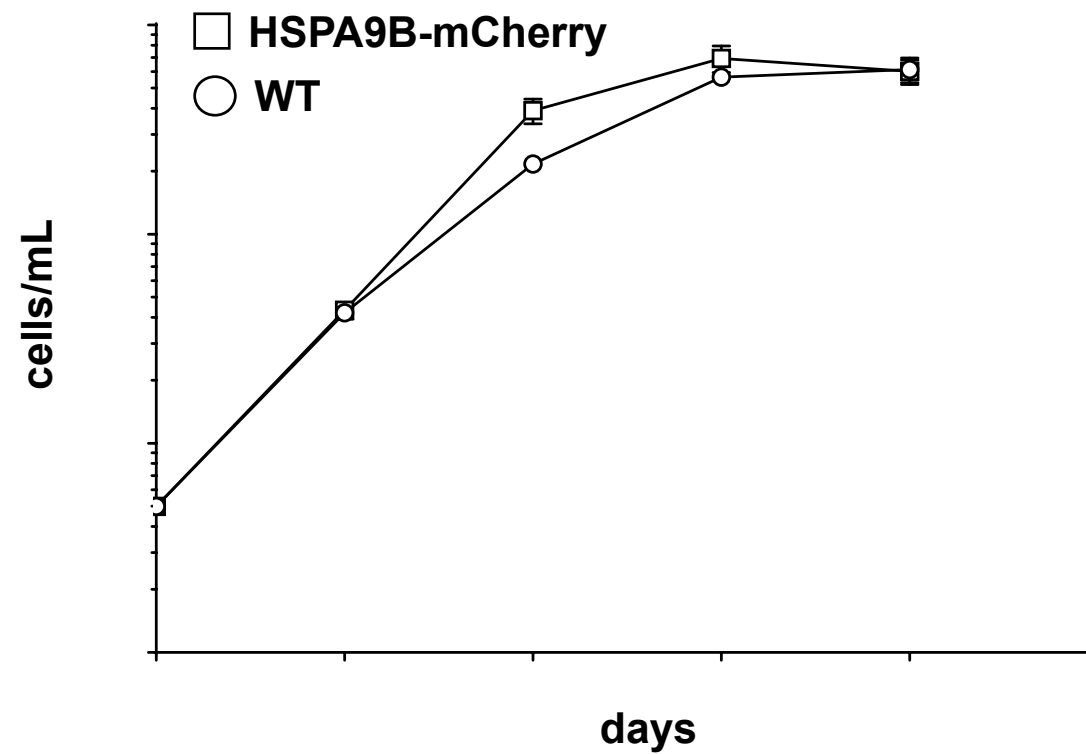

Supplement: Additional file 4: Figure S4. — Growth curves of L. donovani transgenic lines. Parasite concentration of (a) GFP-pXG mock and GFP-HSPA9B-pXG and (b) WT and HSPA9B-mCherry promastigote cultures was measured microscopically by counting cells in a Neubauer chamber daily for 5 days. The mean ± SD of triplicate determinations are shown. (PDF 36 kb) [file 13071_2016_1904_MOESM4_ESM.pdf]

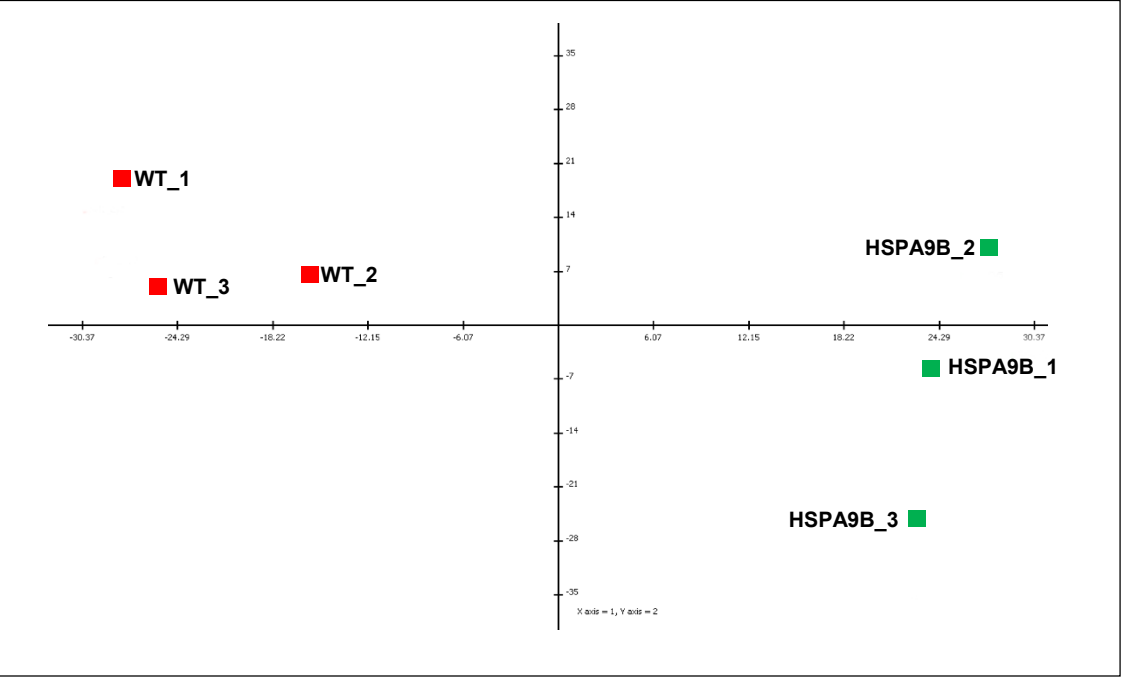

Supplement: Additional file 6: Figure S5. — Principal components analysis (PCA) of WT vs HSPA9B-mCherry 2D-DIGE. Statistical analysis shows the “good” clustering of the biological replicates for each sample. (PDF 41 kb) [file 13071_2016_1904_MOESM6_ESM.pdf]
