# Supplementary material for: A mitochondrial HSP70 (HSPA9B) is linked to miltefosine resistance and stress response in Leishmania donovani
Source: Parasit Vectors. 2016 Dec 1;9:621. doi: 10.1186/s13071-016-1904-8 (PMC5133764; doi:10.1186/s13071-016-1904-8)
Supplement: Additional file 5: Table S1. — Cross-resistance to reference compounds. HSPA9B-mCherry overexpressing lines are significantly more resistant to amphotericin B, antimony (III) and pentamidine isethionate when compared to WT cultures. All the cultures were analyzed in triplicate and data is indicated as mean ± SD of three independent experiments. (PDF 40 kb) [file 13071_2016_1904_MOESM5_ESM.pdf]

Table S1

EC50s	Ld GFP-pXG	Ld GFP-HSPA9B-pXG	LdWT	Ld HSPA9B-mCherry
paromo (uM)	nd	nd	209.26±3.75	205.15±9.29
SbIII (uM)	88.06±5.99	112.6±2.83	81.44±3.93	130.36±8.84
Ampho (nM)	110±10.55	117.5±21.73	117.99±10.16	166.0±3.93
penta (ug/ml)	0.42±0.02	0.41±0.01	0.44±0.07	0.63±0.06
